# Construction and immunogenicity of SARS-CoV-2 virus-like particle expressed by recombinant baculovirus BacMam

**DOI:** 10.1128/spectrum.00959-24

**Published:** 2024-06-25

**Authors:** Hai Trong Nguyen, Darryl Falzarano, Volker Gerdts, Qiang Liu

**Affiliations:** 1 Vaccine and Infectious Disease Organization (VIDO), University of Saskatchewan, Saskatoon, Saskatchewan, Canada; 2 Department of Veterinary Microbiology, Western College of Veterinary Medicine, University of Saskatchewan, Saskatoon, Saskatchewan, Canada; 3 Vaccinology and Immunotherapeutics, School of Public Health, University of Saskatchewan, Saskatoon, Saskatchewan, Canada; Fred Hutchinson Cancer Center, Seattle, Washington, USA

**Keywords:** SARS-CoV-2, virus-like particle, baculovirus BacMam, vaccine, immune response, pseudotyped lentivirus, neutralizing antibody

## Abstract

The pandemic severe acute respiratory syndrome coronavirus 2 (SARS-CoV-2) continues to evolve to give rise to variants of concern that can escape vaccine-induced immunity. As such, more effective vaccines are urgently needed. In this study, we evaluated virus-like particle (VLP) as a vaccine platform for SARS-CoV-2. The spike, envelope, and membrane proteins of the SARS-CoV-2 Wuhan strain were expressed by a single recombinant baculovirus BacMam and assembled into VLPs in cell culture. The morphology and size of the SARS-CoV-2 VLP as shown by transmission electron microscopy were similar to the authentic SARS-CoV-2 virus particle. In a mouse trial, two intramuscular immunizations of the VLP BacMam with no adjuvant elicited spike-specific binding antibodies in both sera and bronchoalveolar lavage fluids. Importantly, BacMam VLP-vaccinated mouse sera showed neutralization activity against SARS-CoV-2 spike pseudotyped lentivirus. Our results indicated that the SARS-CoV-2 VLP BacMam stimulated spike-specific immune responses with neutralization activity.

**IMPORTANCE:**

Although existing vaccines have significantly mitigated the impact of the COVID-19 pandemic, none of the vaccines can induce sterilizing immunity. The spike protein is the main component of all approved vaccines for severe acute respiratory syndrome coronavirus 2 (SARS-CoV-2) due primarily to its ability to induce neutralizing antibodies. The conformation of the spike protein in the vaccine formulation should be critical for the efficacy of a vaccine. By way of closely resembling the authentic virions, virus-like particles (VLPs) should render the spike protein in its natural conformation. To this end, we utilized the baculovirus vector, BacMam, to express virus-like particles consisting of the spike, membrane, and envelope proteins of SARS-CoV-2. We demonstrated the immunogenicity of our VLP vaccine with neutralizing activity. Our data warrant further evaluation of the virus-like particles as a vaccine candidate in protecting against virus challenges.

## INTRODUCTION

The coronavirus disease 2019 (COVID-19) pandemic is caused by a highly transmissible and pathogenic positive-sense, single-stranded RNA coronavirus, severe acute respiratory syndrome coronavirus 2 (SARS-CoV-2) (1). The virus primarily affects the respiratory system, causing sickness with symptoms such as fever, malaise, dry cough, shortness of breath, and respiratory distress (2). SARS-CoV-2 infection is now known to manifest as systemic inflammation, leading to sepsis, acute cardiac injury, heart failure, and multi-organ dysfunction in patients at high risk (3). Globally, more than 775 million confirmed cases and over 7 million deaths have been reported to the World Health Organization as of 31 March 2024 (https://www.who.int/publications/m/item/covid-19-epidemiological-update-edition-166).

The SARS-CoV-2 genome has undergone thousands of mutations since the emergence of the virus (4–6). That leads to the surge of new viral strains, classified as variants of concern (VOCs), possessing varied virulence, transmissibility, and responses to available diagnostics, vaccines, and therapeutics (7, 8). The increasing prevalence of SARS-CoV-2 variants has raised serious concerns regarding possible increased infection severity or failure on the effectiveness of current vaccines thus giving rise to greater challenges to diagnostic and clinical management (7). Vaccination remains the most effective and cost-efficient means for preventing infectious diseases (9). Many different types of vaccines have been developed, including some approved for immunization widely in humans to control SARS-CoV-2 infection in the past 3 years. Although the authorized COVID-19 vaccines are still effective in preventing severe illness, mRNA and adenovirus vector vaccines showed impaired effectiveness and rapid immunity waning against VOCs (10). Therefore, development of more effective, safer, and broad-spectrum vaccines is critically needed to respond to the evolution of SARS-CoV-2 and control COVID-19.

Virus-like particles (VLPs) are self-assembled structures from viral antigens that mimic the three-dimensional morphological structure of authentic virions (11, 12). VLPs have been demonstrated to be very effective against several viral pathogens in humans and thus are considered a highly potential vaccine platform (13). Different approaches have been reported for generating SARS-CoV-2 VLPs (13–16). Recombinant baculovirus-based vaccines are safe and induce humoral and cellular responses (17–19). In addition, baculoviruses have been shown to enhance the immunogenicity of the vaccine antigen, a similar function of an adjuvant (20). In this study, we generated a SARS-CoV-2 VLP using recombinant baculovirus BacMam and evaluated its immunogenicity in a mouse trial.

## MATERIALS AND METHODS

### Cells and culture media

Spodoptera frugiperda (Sf9) cells were propagated at 28°C in Sf-900 II SFM (ThermoFisher Scientific) supplemented with 100 units penicillin and 100 µg streptomycin/mL (MilliporeSigma) and 5% (vol/vol) fetal bovine serum (MilliporeSigma). HEK-293 and HEK-293T cells were cultured and maintained in Dulbecco’s Modified Eagle Medium (DMEM, ThermoFisher Scientific) supplemented with 10% FBS and 100 units penicillin and 100 µg streptomycin/mL (MilliporeSigma) (designated D10 medium) at 37°C in 5% CO_2_ atmosphere.

### Plasmids construction

**Fig 1:**
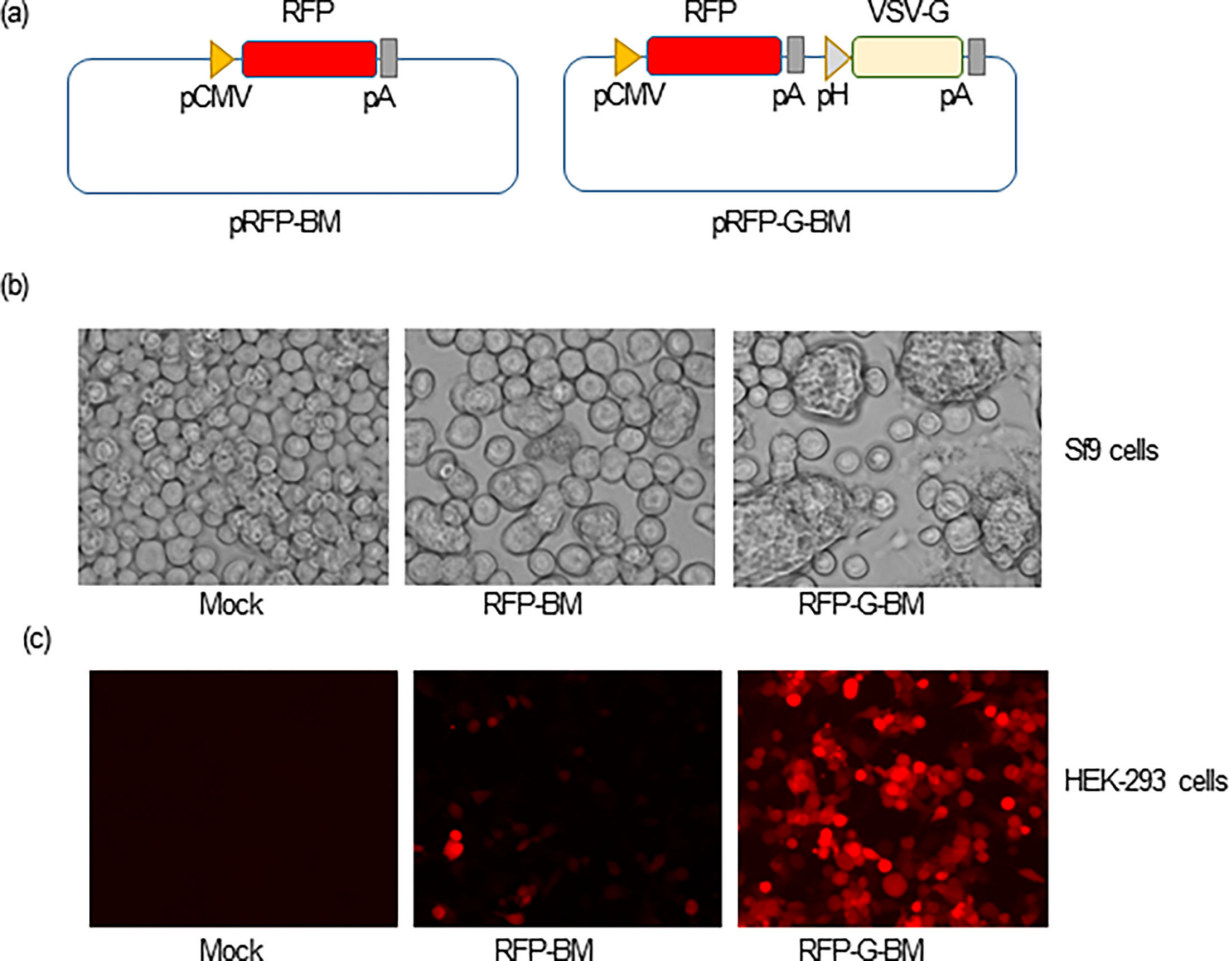
Construction and characterization of RFP-expressing BacMam viruses RFP-BM and RFP-G-BM. (a) Schematic diagram of the pRFP-BM and pRFP-G-BM donor plasmids generating RFP-expressing BacMam viruses. pCMV: Cytomegalovirus promoter; RFP: red fluorescent protein; pA: poly A sequence; and VSV-G: vesicular stomatitis virus glycoprotein. (b) Typical cytopathic effect in Sf9 cells at 48 h post-transduction (hpt). Sf9 cells were transduced with the RFP-BM and RFP-G-BM viruses [multiplicity of infection (MOI) = 1] and observed at 48 hpt by bright-field microscopy. (c) RFP expression in BacMam-transduced HEK-293 cells. HEK-293 cells were transduced with the RFP-BM and RFP-G-BM viruses (MOI = 20) and observed under a fluorescent microscope at 48 hpt.

To generate baculoviruses (BacMam) capable of expressing a single and multiple proteins in mammalian cells and thus could be used directly as an expression system in mammals (21), we first constructed a baculovirus donor plasmid carrying a reporter gene, red fluorescent protein (RFP). A DNA fragment containing CMV promoter followed by the RFP-coding sequence was amplified by PCR with pDsRed-Monomer-C1 (Clontech) as the template and primers CMV-RFP-F and CMV-RFP-R. The PCR product was cloned into the pFastBac1 vector (ThermoFisher Scientific) for generating an RFP-expressing BacMam (assigned as pRFP-BM) (Fig. 1a). To generate a BacMam with the potential of increasing transduction efficiency in mammalian cells, an expression cassette containing vesicular stomatitis virus glycoprotein (VSV-G) coding sequence placed downstream of Polyhedrin promoter and followed by the SV40 poly A signal sequence was amplified by PCR with primer pairs (i) AvrII-pH-F and pH-VSV-G-R, (ii) pH-VSV-G-F and VSV-G-SV40pA-R, and (iii) VSV-G-SV40pA-F and SV40pA-AvrII-R, and cloned into the pRFP-BM plasmid at the AvrII site (assigned as pRFP-G-BM) (Fig. 1a).

**Fig 2:**
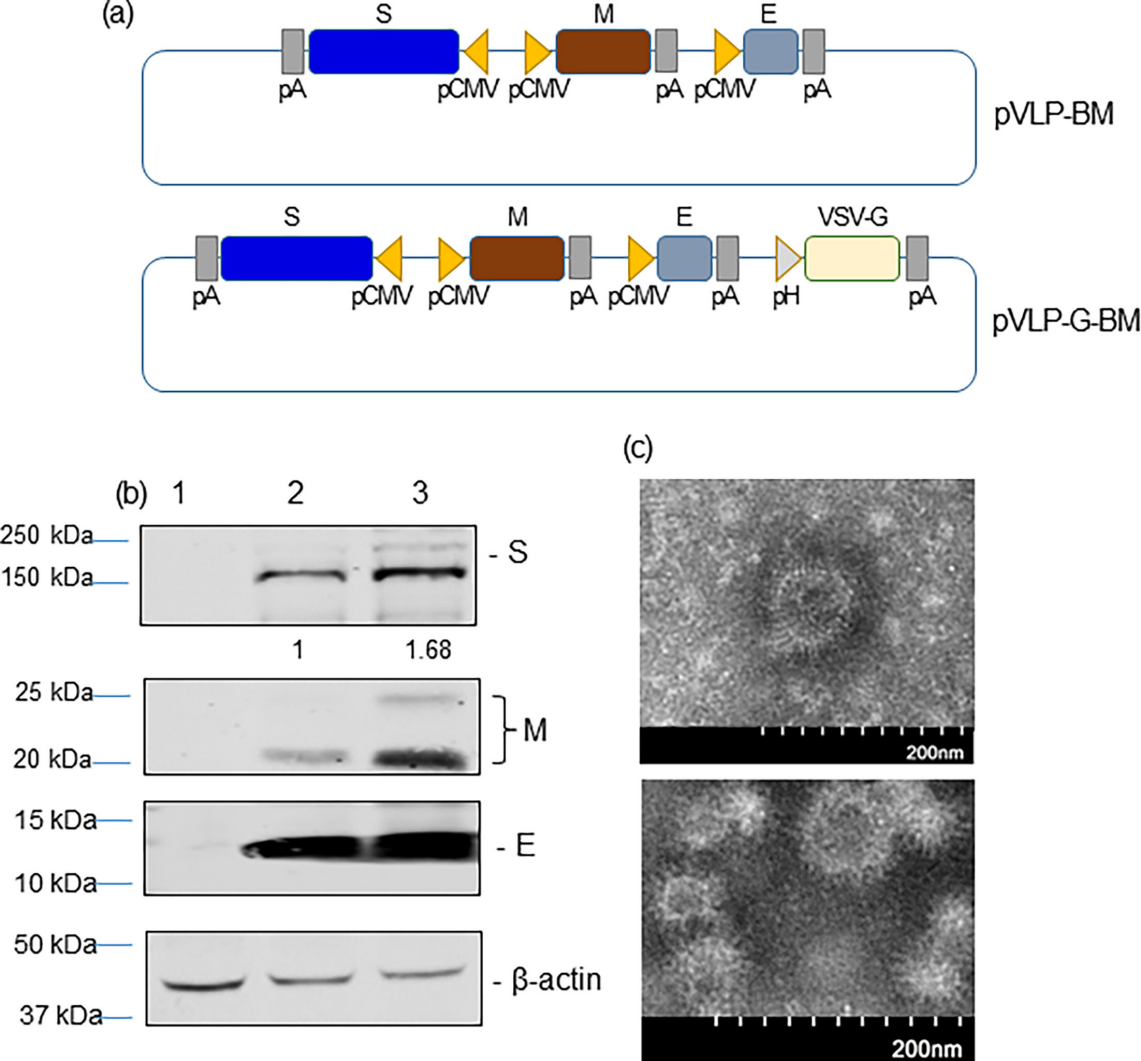
Construction and characterization of SARS-CoV-2 VLP-expressing BacMam viruses VLP-BM and VLP-G-BM. (a) Schematic diagram of the pVLP-BM and pVLP-G-BM donor plasmids. pCMV, Cytomegalovirus promoter; S, M, and E, SARS-CoV-2 Wuhan spike, membrane, and envelope, respectively; pA, poly A sequence; pH, polyhedrin promoter; and VSV-G, vesicular stomatitis virus glycoprotein. (b) Identification of SARS-CoV-2 S, M, and E proteins in BacMam-transduced HEK-293 cells. Non-transduced cells (lane 1), cells transduced with VLP-BM (lane 2), or VLP-G-BM (lane 3) viruses [multiplicity of infection (MOI) = 20] were harvested at 48 hpt for Western blotting with spike-, membrane-, envelope-, or β-actin-specific antibodies. (c) Morphology of SARS-CoV-2 VLP produced in HEK-293 cells. Cells were transduced with VLP-BM virus at an MOI of 20 for VLP purification by sucrose gradient centrifugation at 48 hpt and subjected to transmission electron microscopy.

To construct recombinant donor plasmids for generation of a baculovirus expressing SARS-CoV-2 VLP, coding sequences for spike (S), membrane (M), and envelope (E) proteins of SARS-CoV-2 ancestral Wuhan strain (GenBank accession number NC_045512) were codon optimized for mammalian expression and synthesized (Bio Basic Canada). An intermediate plasmid (pS-BM) was first constructed by inserting the S gene in reverse orientation into the pFastBacTriple1 vector (ThermoFisher Scientific). Subsequently, four DNA fragments containing the M and E genes were amplified by PCR with four pairs of primers, (i) Rsr2-CMV-F and CMV-Cov2-M-R, (ii) CMV-Cov2-M-F and pP10-CMV-R, (iii) pP10-CMV-F and CMV-Cov2-E-R, and (iv) CMV-Cov2-E-F and MfeI-Cov2-E-R, then assembled into the pS-BM plasmid using GenBuilder Plus Cloning Kit (Genscript) to generate pVLP-BM (Fig. 2a). Each gene was placed downstream of CMV promoter and followed by a poly A signal sequence. HSV TK poly A signal sequence was inserted downstream of the S and M genes, while SV40 poly A signal sequence was cloned downstream of the E gene. To generate a second version of SARS-CoV-2 VLP BacMam, a donor plasmid was constructed by assembling PCR fragments containing the VSV-G-expressing cassette as described above into the pVLP-BM plasmid and assigned as pVLP-G-BM (Fig. 2a).

Human angiotensin-converting enzyme 2 (ACE2) cDNA in a plasmid was obtained from Sino Biological Inc. ACE2 coding sequence with a carboxyl V5-polyhistidine tag was cloned into the pEB4.6 episomal vector with a puromycin-resistant gene to allow selection of cells that stably express ACE2 protein after transfection (22). pHAGE-CMV-Luc2-IRES-ZsGreen-W is a lentiviral backbone plasmid expressing both luciferase and GFP reporters and was obtained from BEI Resources (NR-52516) (23). A packaging plasmid psPAX2 was a gift from Didier Trono (Addgene plasmid #12260). Plasmid expressing the SARS-CoV-2 D614G spike protein with a C-terminal 19 amino acid deletion pcDNA-SARS-CoV-2-D614G-D19 WT was kindly provided by Dr. Bo Meng (24). Phusion High-Fidelity DNA Polymerase (ThermoFisher Scientific) was used for PCR amplification. Primers and plasmids used in this study are listed in Tables 1 and 2, respectively. All constructed plasmids were verified by PCR targeting the insertion junctions, restriction enzyme digestion, and DNA sequencing.

**TABLE 1:** List of primers for plasmid construction

No.	Primers	Sequences (5′ - 3′)	References
1	CMV-RFP-F	GGCGCGGATCGCTAGCGAATTCGGATCCACATTGATTATTGACTAGTTATT	This study
2	CMV-RFP-R	GATGACGTCCTCGGTGTTGTCCATGGTGGCGCCAGTAAGCAGTGGGTTCTC	
3	AvrII-pH-F	CATGTCTGGATCTGATCACTGCTTGAGCCTAGGCCGGAATATTAATAGATCATG	
4	pH-VSV-G-R	GTACAAAAGGCACTTCATGAATTCCGCGCGCTTCGGAC	
5	pH-VSV-G-F	GCGCGCGGAATTCATGAAGTGCCTTTTGTACTTAGC	
6	VSV-G-SV40pA-R	CTAGTACTTCTCGACTTACTTTCCAAGTCGGTTCATC	
7	VSV-G-SV40pA-F	CCGACTTGGAAAGTAAGTCGAGAAGTACTAGAGGATC	
8	SV40pA-AvrII-R	CTAGATTTCACTTATCTGGTTCGGATCTCCTAGGCTCAAGCAGTGATCAGATCC	
9	Rsr2-CMV-F	CATACCGTCCCACCATCGGGCGCGGATCCCGGTCCGACATTGATTATTGACTAGTTATT	
10	CMV-Cov2-M-R	GCTATCGGCCATGGTGGCGCCAGTAAGCAGTGGGTTCTC	
11	CMV-Cov2-M-F	CTGCTTACTGGCGCCACCATGGCCGATAGCAACGGGAC	
12	pP10-CMV-R	GTCAATAATCAATGTGGTGATATCGTGTCGGGCC	
13	pP10-CMV-F	CGACACGATATCACCACATTGATTATTGACTAGTTATT	
14	CMV-Cov2-E-R	GCTGTACATGGTGGCGCCAGTAAGCAGTGGGTTCTC	
15	CMV-Cov2-E-F	GCTTACTGGCGCCACCATGTACAGCTTCGTGTCCGAGG	
16	MfeI-Cov2-E-R	CCATTATAAGCTGCAATAAACAAGTTAACAACAACAATTGCATTCATTTTATGTTTCAGG	

**TABLE 2:** Plasmids for generating BacMam, pseudotyped lentiviruses, and ACE2-expressing cells

No.	Plasmids	Descriptions	References
1	pRFP-BM	pP10 - pCMV - RFP-V5-His_6_ - SV40 poly A in pFastBac1 vector	This study
2	pRFP-G-BM	pP10 - pCMV - RFP-V5-His_6_ - pPH - VSV G - SV40 poly A in pFastBac1 vector	
3	pVLP-BM	HSV TK poly A (anti-sense) - *Sph*I - spike (S) (anti-sense) - pCMV (anti-sense) - pP10 (anti-sense) - pPH - pCMV - M - HSV TK poly A - pP10 - pCMV - E - SV40 poly A in pFastBacTriple1 vector	
4	pVLP-G-BM	HSV TK poly A (anti-sense) - *Sph*I - spike (S) (anti-sense) - pCMV (anti-sense) - pP10 (anti-sense) - pPH - pCMV - M - HSV TK poly A - pP10 - pCMV - E - SV40 poly A - pPH - VSV G - SV40 poly A in pFastBacTriple1 vector	
5	pACE2	pCMV—human angiotensin I converting enzyme (peptidyl-dipeptidase A) 2 (ACE2) cDNA-V5-His_6_ in pEB4.6 episomal vector	
6	pSARS2-S	pcDNA-SARS-CoV-2-D614G-S-D19 in pcDNA3.1	(24)
7	psPAX2	A packaging plasmid for second- or third-generation lentiviral vectors and envelope expressing plasmid	Addgene (12260)
8	pLuc2-GFP	Lentiviral backbone plasmid—Luc2, ZsGreen	BEI resources (NR-52516)

### Generation of recombinant BacMam viruses

To generate recombinant baculoviruses, bacmid DNA was extracted from *E. coli* DH10Bac (ThermoFisher Scientific) that were transformed with the donor plasmids. Sf9 cells in a T25 flask were transfected with 3 µg of the bacmid DNA using Cellfectin II Reagent (ThermoFisher Scientific). Culture media containing the viruses were harvested 4–6 days post-transfection (dpt). Virus titers were determined as infectious focus unit (ifu) using the BacPAK Baculovirus Rapid Titer Kit (Takara). Viruses were amplified by infecting Sf9 in suspension culture at a multiplicity of infection (MOI) of 0.1 and harvested at 4 days post-infection (dpi). To purify the virus from infected cell culture, cell debris was removed by centrifugation at 2,000 rpm for 10 minutes. The supernatant was layered over a 27% sucrose cushion in TSE buffer and centrifuged at 24,000 RPM for 75 minutes. The virus pellet was re-suspended in phosphate-buffered saline (PBS, pH 7.5) and centrifuged at 27,000 RPM for 150 minutes and then the final pellet was resuspended in PBS. The purified virus was titrated and stored at −80°C until further use.

### Transduction and fluorescent microscopy

HEK-293 cells were pre-seeded overnight in a 12-well plate to reach 60%–70% confluence then incubated with the BacMam viruses in PBS or DMEM for 2–3 h at 28°C. The transduction mixtures were subsequently replaced with 1 mL of D10 medium supplemented with 5 mM sodium butyrate. The cells were observed under a fluorescent microscope (Zeiss Axioplan 2) at 48 h post-transduction and/or harvested in an SDS sample buffer for protein identification by Western blotting.

### Western blotting

Cell lysates were subjected to sodium dodecyl sulfate-polyacrylamide gel electrophoresis (SDS-PAGE) under reducing conditions. After transferring onto nitrocellulose membranes (Millipore), the membranes were blocked with 3% BSA in PBST (PBS + 0.1% Tween 20) at room temperature (RT) for 1 h and incubated with a primary antibody at 4°C overnight. After five washes with PBST, the membranes were subsequently incubated with the appropriate infrared dye-labeled secondary antibodies for 1 h at room temperature, followed by five washes with PBST, and then scanned with Odyssey CLx Imaging System (Li-Cor Biosciences). Rabbit anti-SARS-CoV-2 Spike Protein S1/S2 (ThermoFisher Scientific), rabbit anti-SARS-CoV Membrane (M) Protein (Rockland), rabbit anti-SARS-CoV-2 Envelope (E) Protein (ThermoFisher Scientific), mouse anti-His-tag (Qiagen), and mouse anti-β-actin (Cell Signaling Technology) antibodies were used as primary antibodies. IRDye 680 goat anti-rabbit IgG (Li-Cor Biosciences) and IRDye 800 goat anti-mouse IgG (Li-Cor Biosciences) were utilized as secondary antibodies.

### Transmission electron microscopy

Purified SARS-CoV-2 VLPs were subjected to negative staining and examined by transmission electron microscopy (TEM). Specifically, a 400-mesh copper grid (Electron Microscopy Sciences) coated with formvar and carbon was placed on a drop of the purified VLP sample and incubated at RT for 1–2 minutes for the particles to adhere, prior to being transferred to a droplet of water for 20 seconds. Subsequently, the grid was placed on a drop of 0.5% PTA stain solution for 1 minute, wicked away the excessive stain, and dried. The grid was then analyzed using a transmission electron microscope, HT7700 (Hitachi High-Tech), and images were acquired through a CCD camera, XR16 (AMT) at the University of Saskatchewan WCVM Imaging Centre.

### Mouse immunogenicity trial

All animal procedures were approved by the Animal Research Ethics Board, University of Saskatchewan (Protocol # 2020-0016). To examine the immunogenicity of SARS-CoV-2 VLP BacMam, BALB/c mice (5–6 weeks old, 8 per group) were intramuscularly injected with PBS or 2.25 × 10^8^ ifu of VLP-G-BM in 50 µL, 25 µL per hind-leg. A second dose of the same vaccine was given after 2 weeks. Vaccinated animals were observed for general health status and reaction at the injection sites. Blood samples were collected before and 14 days post-vaccination (dpv). On day 14 after the second immunization, all mice were humanely euthanized by isoflurane overdose to collect blood and bronchoalveolar lavage (BAL) as per an established protocol (25).

### ELISA

Clear round-bottom Immuno 96-well plates (ThermoFisher Scientific) were coated with 100 µL of purified SARS-CoV-2 S1 protein (2 µg/mL) at 4°C overnight. The plates were blocked with 150 µL of 1% Bovine Serum Albumin (MilliporeSigma) in TBST (Tris-buffered saline +0.1% Tween 20) for 1 h. Subsequently, 100 µL of twofold diluted mouse serum at an initial dilution of 1:200 was added to the coated wells and incubated for 2 h. Then Biotin-labeled goat anti-mouse IgG (ThermoFisher Scientific) was added and incubated for 1 h. Subsequently, the wells were incubated with Streptavidin Alkaline Phosphatase (Jackson Immunoresearch Laboratories) for 1 h at RT. The plates were washed five times with TBST between incubation steps. Finally, p-Nitrophenyl Phosphate (ThermoFisher Scientific) diluted in DE buffer (10.5 mM Diethanolamine, 0.5 mM Magnesium Chloride) was added for colored product development and absorbance was measured at 450 nm with a subtracted reference at 490 nm using a SpectraMax microplate reader (Molecular Devices).

### Creation of ACE2-expressing HEK-293 cells

HEK-293 cells in a six-well plate were transfected with 2.5 µg of the ACE2-V5-polyhistine expressing plasmid using JetPEI (Polyplus) and the culture medium was replaced after 4 h. On the next day, 2 µg/mL Puromycin was added to the cell culture medium, and the concentration of the antibiotic was gradually increased to 10 µg/mL after 4 days of transfection and onwards. The cells that survived under the selection medium were sub-cultured and ACE2 protein expression was confirmed by Western blotting using an anti-His-tag antibody.

### Generation of SARS-CoV-2 spike pseudotyped lentivirus, confocal microscopy, and luciferase assay

Pseudotyped lentivirus with both luciferase and green fluorescent protein (GFP) reporters was generated as described previously (23, 26). In brief, HEK-293T cells in six-well plate were co-transfected with 0.34 µg of spike-expressing plasmid pcDNA-SARS-CoV-2-D614G-S-D19 WT (24), 1 µg of lentiviral backbone plasmid pHAGE-CMV-Luc2-IRES-ZsGreen-W (23), and 0.66 µg of packaging plasmid psPAX2 plasmids using the JetPEI transfection reagent. Cell culture medium was replaced at 16 hpt and supernatants containing SARS-CoV-2 pseudovirus were collected at 48 and 72 hpt, filtered through a 0.45-µm filter, aliquoted, and stored at −80°C.

HEK-293-ACE2 cells seeded in 96-well cell-culture plate (~5 × 10^4^ cells/well) pre-treated with Poly-L-lysine (MilliporeSigma) were infected with the SARS-CoV-2 pseudovirus in the presence of 5 µg/mL Polybrene (MilliporeSigma) to minimize charge-repulsion between the virus and cells (27). The lentiviral pseudovirus stock volume was used to sufficiently produce >2 × 10^6^ relative light units (RLUs) per well in a 96-well plate for achieving a signal of 10^4^-fold above the background luciferase activity of mock-infected controls to give a dynamic range sufficient for use in neutralization assay (28). The infected cells were harvested at 48 h post-infection (hpi) and luciferase assay was performed using Luciferase Reporter Assay System (Promega) in a GloMax 20/20 Luminometer according to the manufacturer’s instructions.

HEK-293-ACE2 cells cultured in two-well chamber slides (ThermoFisher Scientific) were similarly infected with the SARS-CoV-2 pseudovirus. At 48 hpi, the infected cells were fixed, and the nuclei were stained with 300 nM DAPI (MilliporeSigma) for 10 minutes at RT. After applying a mounting medium (ProLong Diamond Antifade Mountant, ThermoFisher Scientific), the slides were covered with a coverslip and dried in the dark for 24 h before examination of GFP expression by confocal microscopy.

### Pseudotype virus neutralization assay

Neutralization assay was performed as described previously with minor modifications (23, 29, 30). In brief, pooled serum samples of VLP-G BM- and PBS-injected mice at 28 dpv diluted 1:20 in D10 medium were incubated with pseudovirus for 1 h at 37°C, then utilized to infect the HEK-293-ACE2 cells in 96-well cell-culture plate. The cells were observed under a fluorescence microscope (Zeiss Axioplan 2) with appropriate settings for GFP expression at 24–48 hpi and then lysed with the Passive Lysis buffer (Promega) at 48 hpi for 30 minutes at RT for luciferase assay. Neutralization activity was determined by the RLUs per well in a 96-well plate of the vaccination group relative to the PBS control group.

### Statistical analysis

All experiments in cell culture were done in triplicates and data were expressed as mean ± *SEM* values. Data analysis was performed using GraphPad Prism 9 and statistical differences were determined by the Student’s *t*-test. Statistical significance was demonstrated as follows: * if *P* < 0.05, ** if *P* < 0.01, *** if *P* < 0.001, **** if *P* < 0.0001, and *NS* if insignificant.

## RESULTS

### Generation and characterization of SARS-CoV-2 VLP BacMam

To explore the potential of a baculovirus-based VLP vaccine against SARS-CoV-2, we first generated recombinant baculoviruses expressing an easily detectable reporter for testing the capability of expressing foreign proteins and transduction/infection efficiency of this system *in vitro*. To this end, we first constructed two baculovirus donor plasmids containing RFP gene under the control of CMV promoter followed by a poly A sequence with and without incorporation of VSV-G-coding sequence under the control of Polyhedrin promoter assigned as pRFP-BM and pRFP-G-BM, respectively (Fig. 1a). Recombinant baculoviruses expressing RFP protein in mammalian cells were successfully generated by transfecting Sf9 cells with the bacmids. Typical cytopathic effects (CPEs) in the form of cell-to-cell fusion and cell swelling were clearly observed in Sf9 cells after 2 days of infection with these two BacMam viruses and this phenotype was stronger in the RFP-G-BM-infected cells possibly due to membrane-fusion activity of VSV-G protein (Fig. 1b). Expression of RFP was clearly observed 2 days post-transduction with noticeably higher number of RFP-positive HEK-293 cells transduced with the RFP-G-BM containing VSV-G (Fig. 1c). Our results confirmed that incorporation of VSV-G in recombinant baculoviruses significantly enhances transduction efficiency of the virus into mammalian cells and results in a significant improvement on protein expression (31, 32). In addition, our recombinant BacMam viruses showed great capability of infecting/transducing several mammalian cell lines, including HEK-293, HEK-293T, Vero 76, Vero E6, and Huh-7, as evidenced by RFP expression observed under a fluorescent microscope (data not shown).

We then utilized this BacMam system to produce SARS-CoV-2 VLPs. Two BacMam viruses expressing SARS-CoV-2 S, M, and E proteins, a minimal requirement for coronavirus VLP formation (33), with and without VSV-G were generated, assigned as VLP-BM and VLP-G-BM, respectively. The arrangement of SARS-CoV-2 structural protein-coding sequences in the BacMam donor constructs is shown in Fig. 2a. The expression of SARS-CoV-2 S, M, and E proteins was confirmed by Western blotting using protein-specific antibodies. The levels of β-actin were also determined as protein loading controls. Higher levels of SARS-CoV-2 S, M, and E protein expression in HEK-293 cells transduced with the VLP-G-BM were observed compared with those transduced with the VLP-BM (Fig. 2b). Of note, the M protein appeared as two bands in Fig. 2a, which could be resulted from post-translational modifications and/or multiple conformations of the M protein (34–36). The formation of SARS-CoV-2 VLPs in HEK-293 cells transduced with the VLP-G-BM virus was demonstrated by transmission electron microscopy with negative staining (Fig. 2c). Our VLPs displayed corona-like morphology with round shape, spike-like structures decorated on the surface, and the average diameter fell around 85.2 ± 19.8 nm, which is in accordance with previously reported SARS-CoV-2 VLPs (15) and SARS-CoV-2 particles (37). These results showed that transduction of our VLP-G-BM virus led to the formation of SARS-CoV-2 VLP in mammalian cells.

### Production of SARS-CoV-2 VLP BacMam virus

For the production of the VLP BacMam, we first adapted Sf9 cells in suspension culture and then infected the cells with VLP-G-BM at an MOI of 1. The culture medium of infected cells was harvested 3–4 days post-infection and the recombinant VLP BacMam was purified and concentrated by ultra-centrifugation and stored at −80°C. The titer of the purified VLP BacMam virus was 1.25 × 10^10^ ifu/mL.

### Immunogenicity of SARS-CoV-2 VLP BacMam virus in mice

**Fig 3:**
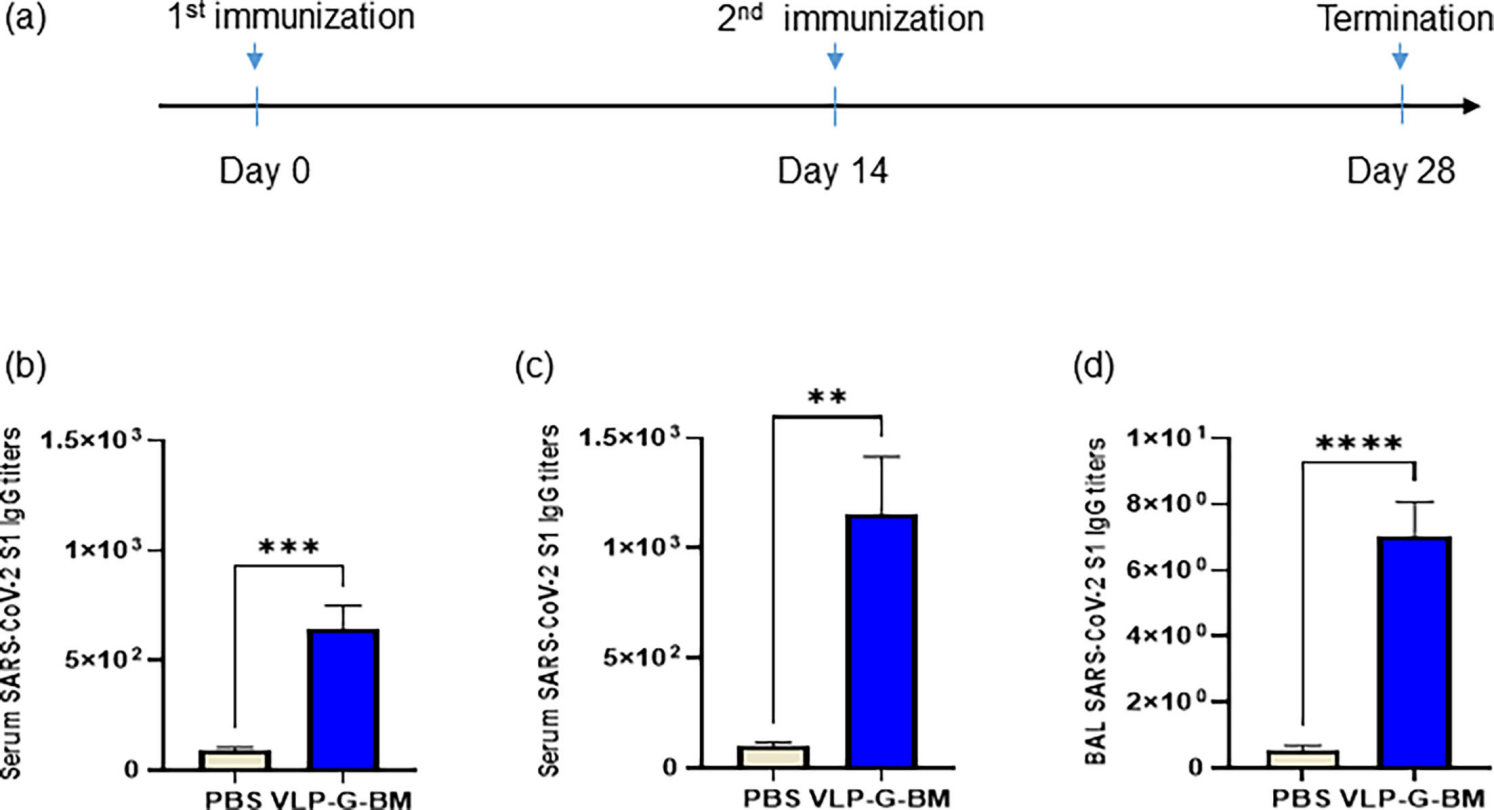
VLP BacMam induced spike-specific immune response in mice. (a) Immunization schedule. BALB/c mice (*n* = 8) were intramuscularly injected with PBS or 2.25 × 10^8^ ifu of VLP BacMam and boosted at 14 days post-vaccination (dpv). Serum samples were collected 14 days after each immunization. Mice were euthanized at 28 dpv to collect bronchoalveolar lavage (BAL) samples. (b, c, and d) SARS-CoV-2 S1-specific IgG titers in sera were determined by ELISA after primary (b) and boosting (c) immunization. SARS-CoV-2 S1-spcific IgG titers in BAL after two immunizations were also determined (d).

To evaluate the immunogenicity of our baculovirus-based VLP vaccine in mice, VLP-G-BM at 2.5 × 10^8^ ifu per mouse was injected intramuscularly and boosted with the same dose of vaccine at two weeks post-primary immunization (Fig. 3a). No overt adverse clinical events were observed in the animals throughout the experiment following vaccination and no body weight loss was recorded (data not shown), demonstrating the safety of the vaccine.

The immune response to SARS-CoV-2 spike S1 protein was examined in sera and bronchoalveolar lavage (BAL) after 14 and 28 days post-vaccination of the VLP BacMam vaccine. Mice vaccinated with the VLP BacMam showed significantly higher serum IgG levels on day 14 after primary immunization (Fig. 3b) and further increased on day 28 after the boosting shot (Fig. 3c), albeit the increase did not reach statistical significance. In addition, the VLP-G-BM induced IgG antibody production in the BAL of vaccinated animals on 28 dpv (Fig. 3d). These results demonstrated that the VLP BacMam vaccine was safe and capable of inducing spike-specific immune responses in sera and the lung after intramuscular immunization.

### VLP BacMam induced neutralization against SARS-CoV-2

**Fig 4:**
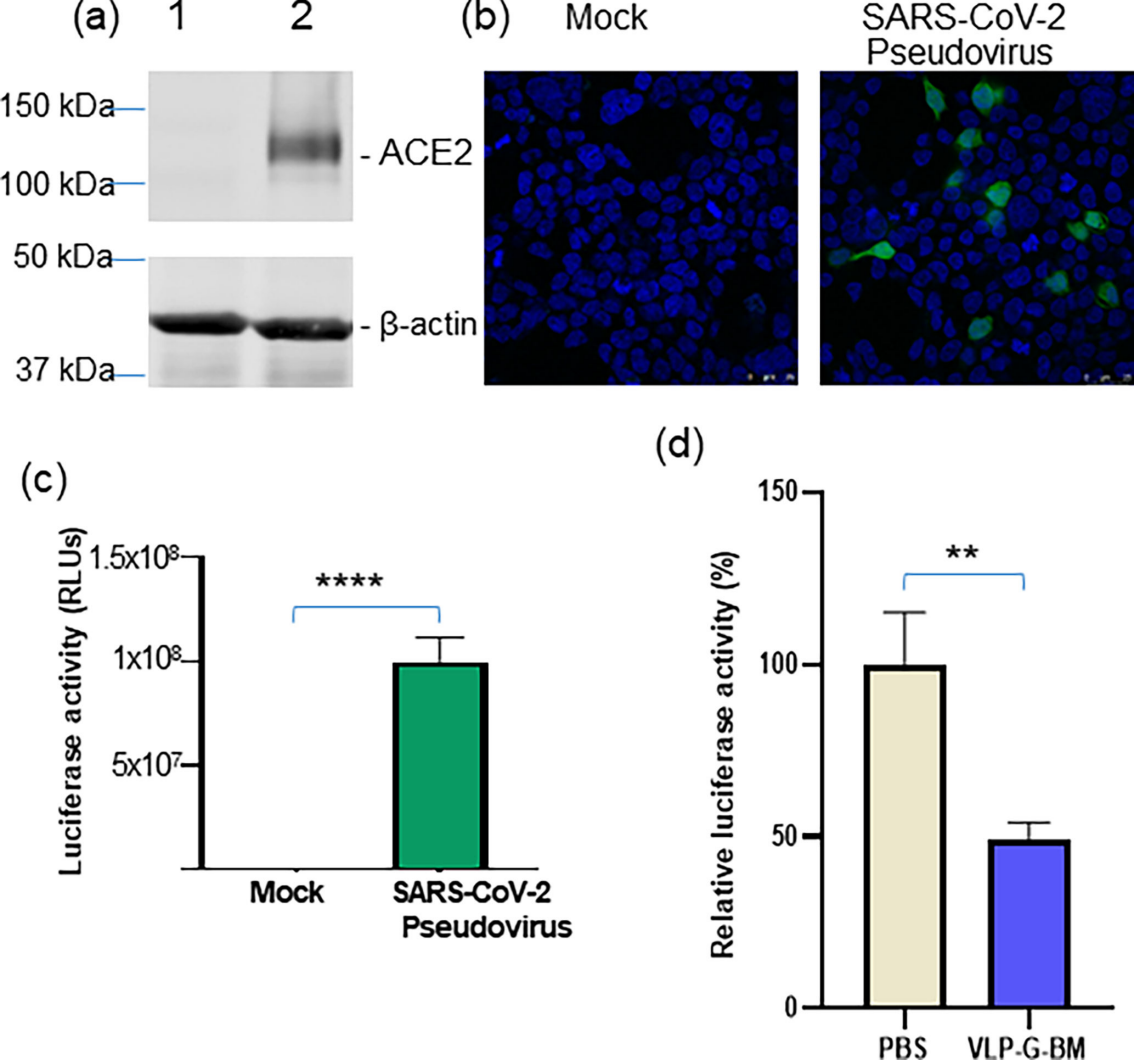
VLP BacMam induced neutralization activity against SARS-CoV-2 spike pseudotyped lentivirus. (a) Identification of ACE2 protein expressed in ACE2-expressing HEK-293 (HEK-293-ACE2) cells. HEK 293 cells were transfected with pACE2-V5-His tag plasmid and selected with Puromycin. Survival cells under selective pressure were sub-cultured and the polyhistine-tagged ACE2 protein expression was demonstrated by Western blotting with an anti-His-tag antibody. The levels of β-actin were demonstrated by an anti-β-actin antibody. Lanes: 1, HEK-293 cell lysate; 2, HEK-293-ACE2 cell lysate. (**b and **c) Reporter expression by SARS-CoV-2 spike pseudotyped lentivirus. HEK-293-ACE2 cells were transduced with mock, or SARS-CoV-2 spike pseudotyped lentivirus virus expressing both GFP and luciferase reporters. Cells were harvested at 48 hpt to demonstrate GFP expression by confocal microscopy (b) and luciferase activity by luciferase assay (c). (d) Neutralizing activity of VLP BacMam-vaccinated mouse sera against SARS-CoV-2 spike pseudotyped lentivirus transduction. SARS-CoV-2 spike pseudotyped lentivirus was treated with PBS- or VLP BacMam-injected mouse sera at a dilution of 1:20 and used for transducing HEK-293-ACE2 cells. The cells were harvested at 48 hpt for luciferase assay. The luciferase activities in the PBS group were set to 100.

To evaluate the capability of inducing neutralization against SARS-CoV-2 of our VLP BacMam vaccine, we produced a pseudotyped lentivirus bearing SARS-CoV-2 spike protein with GFP and luciferase reporters. For efficient infection of the SARS-CoV-2 pseudotyped virus, we also generated an ACE2-expressing cell line (HEK-293-ACE2). The stable expression of ACE2 protein in HEK-293-ACE2 cells was confirmed by Western blotting (Fig. 4a). HEK-293-ACE2 cells infected with the SARS-CoV-2 pseudotyped virus displayed green fluorescence determined by confocal microscopy (Fig. 4b). In addition, significantly higher level of luciferase activity was detected in SARS-CoV-2 pseudotype virus transduced cells than mock transduction (Fig. 4c). Importantly, we observed similar neutralization titers of a reference positive serum sample using both the pseudotyped virus and authentic SARS-CoV-2 virus (data not shown), demonstrating the reliability of the pseudotyped virus for use in neutralization assay.

In comparison to PBS-vaccinated animals, sera of VLP BacMam-vaccinated animals at the dilution of 1:20 showed approximately 50% reduction in luciferase activity in HEK-293-ACE2 cells infected with the SARS-CoV-2 spike pseudotyped virus pre-treated with the VLP BacMam-vaccinated mouse sera (Fig. 4d). These results demonstrated that vaccination with our VLP BacMam induced neutralizing antibodies, which could be capable of protecting against SARS-CoV-2 infection.

## DISCUSSION

Many infectious diseases have emerged or re-emerged over the past few decades. These diseases have led to millions of deaths and caused an enormous slowdown in the global economy (38). The COVID-19 pandemic, caused by highly transmissible SARS-CoV-2, is the most recent example of those deadly infections and has spread all over the globe within a short period, infecting hundreds of millions of people and resulting in millions of deaths. The global spread and impacts of COVID-19 had provoked an urgent need for preventative approaches through vaccinations (39), which has greatly contributed to the overall success in controlling the disease globally so far. However, none of the existing vaccines can induce sterilizing immunity. Because the spike protein is the main component of the approved vaccines due primarily to its ability to induce neutralizing antibodies (40), how to best conserve its authentic conformation in the vaccine formulation plays a critical role in inducing effective immunoprotection. To this end, numerous approaches have been explored, such as locking the spike protein in a prefusion state by introducing multiple point mutations (41, 42). In this work, we expressed the spike protein as part of a virus-like particle and evaluated its immunogenicity.

Recombinant baculoviruses have been reported previously as a safe and effective platform for gene delivery and vaccine development against important viral pathogens (17–19, 31, 43). Engineering mammalian promoters into the baculovirus genome (BacMam) has allowed protein expression in mammalian cells as an additional platform feature (44). In this work, we constructed a recombinant BacMam expressing SARS-CoV-2 structural proteins spike, membrane, and envelope. Transmission electron microscopy experiment showed the formation of VLPs in mammalian cells after BacMam transduction with the morphology and size being very similar to those of authentic SARS-CoV-2 virions. Immunogenicity study showed that the VLP BacMam elicited the production of SARS-CoV-2 spike-specific IgG antibody after intramuscular injections in mouse sera and mouse lungs. More importantly, we showed that the sera of VLP BacMam vaccinated mice inhibited the transduction of a SARS-CoV-2 spike pseudotyped lentivirus, indicating the presence of neutralizing antibodies. However, the neutralizing titer was quite low. We envision that optimizing vaccine dose, vaccination schedule, as well as delivery routes may increase the immunogenicity and neutralizing titers.

The spike protein is the main component of the approved vaccines due primarily to its ability to induce neutralizing antibodies (40). Unfortunately, there have been constant mutations in the spike protein, resulting in altered antigenicity and thus reduced efficacy and rapid immunity waning of the vaccines developed against previous spike sequences (45). Therefore, inclusion of more conserved viral components has been proposed to provide broader protection (40). Another consideration when it comes to improving the existing vaccines is to induce cross-protective T-cell responses, which are important for preventing severe COVID-19 disease (45). In this regard, the M and E proteins have become good candidates due to their high-level conservation and the identification of multiple T-cell epitopes in these two proteins (40). Although the VLP constructed in this study expresses both M and E proteins, we did not characterize the immune responses to these two proteins as we focused on evaluating spike-specific immune responses and the neutralizing activity. As more research has indicated the importance of cross-reactive T-cell responses in providing broad protection and preventing severe illness, future work should be directed toward characterizing the immune protection conferred by M and E proteins.

In conclusion, we demonstrated the formation of SARS-CoV-2 VLP by a recombinant BacMam virus and its capability of inducing immune responses in sera and the lungs in mice after immunization. These results warrant further investigation to evaluate the protective efficacy of the VLP vaccine against viral challenges.
